# Abstract of the Proceedings of the Buffalo Medical Association

**Published:** 1855-07

**Authors:** Sanford B. Hunt


ART. IV.—*Abstract of the Proceedings of the Buffalo Medical Association.*

TUESDAY, June 5, 1855.

The Association met at the office of Dr. Strong.

Present—Drs. Strong, Samo, Gould, Wilcox, Burwell, Gray, Flint, Underhill, Hunt, Hubbard, Richards, Whitney, Wyckoff, Treat, Rochester, Newman, Nelson, Newell, Root, Gray, Jeyte.

Minutes of preceding meeting were read and approved.

Dr. J. C. Lay was elected to membership.

*Dr. Nelson* called the attention of the association to the necessity of care in prescribing, and after relating a case where an important error had occurred, read the following paper on

*Prescribing.*—There is an old adage that there are two ways of doing every thing, a good and a bad; and as in the ordering of medicine we cannot be too particular, I hope I shall be pardoned for offering the following few remarks on prescribing:

It is essentially necessary that the patient's name should be placed upon the prescription, together with the date, and bearing the initials of the attending physician. Many may reply that in this "go-a-head" country we cannot wait to attend to these minor things; that there is no use for the patient's name, because there cannot be a mistake about the individual, and that the directions are given verbally to save time. I say there is every necessity, for the following reasons: Two or more persons may be sick in the one house, afflicted with different diseases, or requiring different treatment; the precaution is indispensable that their prescriptions shall be marked so that the labels on their medicine may be distinguished for each patient; thus there will not be any mistake in exhibiting the proper dose, and the proper medicine to each person.

Again: Two individuals may call together at your office for advice, you may happen to order them both either mixtures or pills, but of an opposite character; they take them to the apothecary to be compounded, unless the greatest care is used the prescriptions may be exchanged and a very serious result follow.

A medical man should never deliver a prescription to a patient without the signature of his initials, as a voucher for its authenticity and accuracy, and his responsibility.

The apothecary, also, in copying the prescription, should enter the patient's name and the directions for using, for how often may a prescription be ordered to be repeated, and a mistake occur in the number, when by having the number and the name it is impossible that an error could occur.

Apothecaries should never repeat a medicine without an order from the physician, because the continued use of a remedy which has been of service so far, may become injudicious, or the physician may wish to make some alteration.

While upon this subject I would wish to draw the attention of the profession to "consultations" between two or more physicians, on a patient.

The physicians having finished their consultation and agreed upon the treatment, the attending medical man should write the prescription, which should be signed by all the medical men in that council, beginning with the senior and ending with the attendant; that is their voucher of agreement and responsibility. They should then return to the room of the patient, and the attendant, or senior physician at his request, deliver their opinion upon the case: it is the patient's or his friends' right to demand it, and without it the counsel are not entitled to their fee. The prescription should then be left with the family and forwarded to some responsible apothecary—the attending physician should not put up the medicine unless there are no apothecaries in the place. But here the responsibility of the counsel does not terminate: a set time ought to be appointed when they should meet again, having become separately and collectively answerable for the case, they should not be debarred from again seeing the patient, unless some occurrence should take place by which it would be impossible for the parties to agree, and thus continue their attendance till the case terminates.

*Dr. Hunt* was called, a few weeks since, to see a child aged ten months, which had fallen a distance of ten or twelve feet, striking its head against the curb of a cistern. On arriving about 1, P. M., he found that the child, which had been comatose for a time, was then conscious. It was pale; pulse slow and feeble; vomiting occasionally; extremities cold; pupils of eyes responded to light. Just behind the right frontal protuberance was a large and very marked depression of the frontal and part of the parietal bones, extending from the anterior fontanelle downward. The bone was indented to the depth of half an inch in its centre. The whole diameter of the depression was about two by three inches. Owing to tumefaction about the fontanelle, it was impossible to say whether fracture existed there, but in all other parts it was evident that the bone was merely depressed, not broken.

Directed cold to the head, warm foot bath, a cathartic, and entire quiet in a darkened room.

6, P. M. Found the patient out doors in its mother's arms, bright and cheerful. The depression was much less than at noon, the bone having, as was anticipated, sprung to place by its own elasticity. The depression grew gradually less, day by day, until the tumefaction disappeared, when it was found that a triangle of bone, the apex of which was at the fontanelle, the base extending downward and forward for an inch, was entirely fractured and depressed.

Saw the case every day or two for a fortnight, and after that, owing to absence, lost sight of it. The last time it was seen it was apparently suffering neither inconvenience or injury from the accident, and no treatment was recommended, preferring to wait the natural issue of the case.

*Dr. Flint* mentioned the presence of an unusual number of cases of intermittent fever at the hospital.

*Dr. Treat* had seen more intermittent fever this spring than any season before for twelve years. Most of his cases had occurred on the outskirts of the city. Had seen also two or three cases of dysentery of a mild form, within the last few days.

*Dr. Wyckoff* had treated an unusual number of cases of pneumonia within the last fortnight, a fact which excited his attention, as during the winter and spring he had seen but very few, almost no cases. These recent cases had presented a very marked inflammatory type, requiring the lancet. The blood taken from the arm, after standing, was very much buffed and cupped, and the crassamentum very firm. Patients required two or three bleedings.

*Dr. Strong* alluding to an unusual number of cases of diarrhœa reported at the April meeting, and regarded as premonitory of cholera, inquired as to its present prevalence. Only a very few cases were mentioned.

*Dr. Whitney* (dentist) reported a case of salivary calculus of unusual size, occurring in a woman who had lost her teeth some twenty years' previously. It occupied the sub-maxillary gland of the right side, and formed a prominence under the jaw, interfering with deglutition. Dr. W. removed it by the forceps with the aid of a slight incision. It was found to be one inch long by three-fourths of an inch in diameter. Weight 63 grs.

*Dr. Hubbard* reported three cases of scarlatina, and the same number of erysipelas of medium severity, in which he had employed inunction, as recommended in the Journals some time since. He thought very favorably of it as a remedy; the children affected liked it, and called for its repetition. It seemed to allay their discomfort.

*Dr. Rochester*, after mentioning a case of unusually mild scarlatina, described a very well marked and fully developed case of measles, occurring in a child five months old. The child had not been out doors, neither was there any measles in the neighborhood. The mother of the child had, however, been on a visit to the country, had visited a family where were some cases of measles, and had returned to the city immediately, a distance of eighteen miles. It was a question of some interest whether the contagion of rubeola could be carried that distance in the clothing.

*Dr. Nelson* reported a case of epilepsy in a woman aged 28 years, Irish; robust; had had the fits for six months, occurring every two or three days. The cause was not evident, but as each fit was preceded by severe pain in the head and bowels, a cathartic was given with the idea that they might be due to fœcal accumulation. For a week after the purging there were no fits, but they soon returned. After the use of a variety of remedies, Dr. N. resorted to the extract of the cotyledon umbilicus, giving five grains twice daily. The fits were then suspended for a fortnight. The effect of the cotyledon was at first to open the bowels, but this ceasing, and the convulsion recurring slightly, the same dose was given three times daily. Still no effect upon the bowels, and the dose was increased to seven and a-half grains three times daily without affecting the discharges. The epilepsy, however, was suspended. At this time, Dr. Nelson being absent for some time, he did not see her for a fortnight, and she was without the medicine for three days, in each of which she had a slight convulsion. There is less headache now, and Dr. N. was much pleased with the effect of the remedy in controlling, if not removing, the disease.

*Dr. Flint* mentioned that any disturbing cause, as a cathartic, would for a while break in upon the epileptic tendency. One of the most valuable contributions to the literature of epilepsy, was that of M. Herpin, who had given the results of the treatment of a large number of cases. M. Herpin uses the oxide of zinc, continued for a long time, and arrives at the conclusion that if the number of convulsions have not exceeded one hundred, much may be hoped from the persevering use of this remedy, but that that number being exceeded, the probability of cure was very much diminished.

*Dr. G. N. Burwell* gave the history of several cases of pneumonia:

J. R., aged 39 years, was taken suddenly ill on Thursday afternoon, May 24. He had a chill, followed by fever; pain in the right side and axilla as high as the nipple; shortness of breath; cough and bloody expectoration.

These symptoms continuing without abatement, Dr. B. was sent for Sunday,

May 27. He had then a warm skin covered with a free perspiration; pulse 96, full and hard; respiration 32; cough troublesome, with an expectoration which was scanty, viscid and bloody; pain in right side felt more especially whenever he coughed or took a deep inspiration; thirst and a general malaise. His rest last night had been very weary, with a good deal of talking in his sleep.

He prescribed calomel and rhubarb for physic, and the tr. verat. viride, in six drop doses, every four hours, unless sickened badly at stomach by it.

May 28. The physic operated well. The veratrum had sickened him after three doses, and had then been discontinued. His chest symptoms existed with very little alteration. Pulse 88, full and hard; there was the same perspiration. His rest at night had been very uneasy. He complained of a good deal of pain and distress through the right lung, but the physical signs were negative. There was feebleness of respiratory murmur, but neither the crepitant rhonchus nor bronchial respiration could be detected.

V. S. ad. 24 oz. The veratrum to be taken and not to be discontinued.

May 29. Dr. Wyckoff in consultation. The blood drawn yesterday was taken in two vessels, a bowl and a tea cup. That in the bowl was covered with a buff half an inch thick—that in the tea cup with one nearly three-fourths of an inch, and well cupped. The crassamentum in either vessel was so firm as to support its own weight.

Pulse to-day 88, still full and hard. Respiration had fallen to 26; skin warm and perspiring; cheeks flushed. Expectoration not so bloody, yet still viscid and rusty. Physical signs negative.

V. S. ad. 22 oz. The veratrum continued.

May 30, A. M. The blood drawn yesterday had precisely the appearance of that drawn the day before. The amount of buff was precisely the same. Pulse 28, but softer than on the previous visits, the patient's pulse faint and he was cool and restless. Respiration 24. Has troublesome paroxysms of coughing. Expectoration viscid but very slightly rusty or yellowish, no red globules in it.

Discontinued the veratrum. Directed eight grains of Dover's powder immediately, to be repeated if the restlessness continued.

P. M. Pulse risen to 96; respiration 30; cheeks flushed; skin warm and less than the usual perspiration.

Repeat the Dover's powder; to take half a grain of ipecac with three grains of the nitrate of potash, every two hours.

May 31. General appearance much improved. Pulse 100, but soft; respiration 26. Expectoration still scanty and viscid; but little heat of skin.

To continue the ipecac and nitrate.

June 1, 11, A. M. He continued to do well until about midnight, when his fever came on with an aggravation of all the symptoms. Pulse 112; respiration 36; is very restless; slept but little after midnight; was quite wandering. The physical examination of the lungs this morning, for the first time, gave decided evidence of the seat of the inflammation. Anteriorily over the upper and middle lobes of the right lung percussion was dull, with well developed bronchial respiration and a fine sub-mucous or crepitant rhonchus.

V. S. ad. 24 oz. No medicine to be taken.

8, P. M. Dr. Wyckoff in consultation. The blood has a decided buffy boat, but not over an eighth of an inch thick. The patient went immediately to sleep after the bleeding, and has since slept perfectly quiet and easy, without much disturbance from cough and delirium. Pulse fallen to 96; respiration 26; skin moist and cool.

No medicine to be given.

June 2. Patient has had the best night since his illness; but little cough or expectoration. Pulse 84; respiratien 24; no fever or thirst. The dullness of percussion noticed yesterday has disappeared, as also the auscultatory signs of inflammation. The respiratory murmur, however, is feeble and on a higher key or pitch than that in the opposite lung.

No medicine to be given.

June 3. Patient fully convalescent. Pulse 72; respiration 18. Feels in every respect comfortable.

June 6. Convalescence continues without interruption.

Case II. W. R., aged 17 years. First seen on Tuesday, May 29. Had a chill, followed by fever, on Friday, May 25. Was perfectly well before. He has now a hot, dry skin; pulse 116, active; respiration 36; has pain in his left shoulder, and a viscid expectoration which is occasionally bloody. His cough is severe, paroxysmal and very troublesome at night. Although there was no doubt of the existence of pneumonia, a careful physical examination did not demonstrate its precise seat.

V. S. ad. 16 oz. He is to take half a grain of opium (in pill) on every severe paroxysm of cough.

May 30. The blood drawn yesterday was taken (as in the last case) in a bowl and tea cup. It was very buffy: that in the bowl being nearly, and that in the cup over, half an inch thick. Took five opium pills last night.

Pulse to-day 116, full and active; respiration 32; expectoration quite abundant, but very viscid and slightly rusty.

V. S. ad. 18 oz. To take the opium pills as directed yesterday. No other medicine directed.

May 31. The blood drawn yesterday was not visibly different from that first taken. Took three of the pills during the night. To-day pulse 104; respiration 36; skin hot; pain in the left shoulder continues.

The physical signs to-day for the first time, point out unequivocally the seat of the inflammation. Percussion is dull over the upper lobe of the left lung anteriorly; auscultation shows a light blowing sound on inspiration and expiration, but without being yet developed into a clear distinct bronchial respiration. No crepitus.

V. S. ad. 16 oz. To take the opium pills if necessary.

June 1. The blood drawn yesterday had only a thin covering of buff, and the crassamentum would not support its own weight without tearing. The patient has had a very good night without taking any opium.

Pulse to-day 100; respiration 32; skin cool. The bronchial respiration is now clearly and fully developed, and a fine crepitus can be heard on a forced inspiration or on coughing.

June 2. The boy is fully convalescent. Pulse 68; respiration 20; has but little cough or expectoration, and neither fever nor pain. The bronchial respiration exists, but without the clearness and distinctness of yesterday. The crepitus is replaced by a mucous rhonchus. The patient wants to eat. Has not taken any medicine for over two days.

June 3. Convalescence uninterrupted. Dr. Wyckoff visited the patient with me daily after the first visit.

As showing the highly inflammatory character of the pneumonia that occurred about this time, I would mention the case of another boy 17 years of age, I was called to see on May 27. He was attacked suddenly on the 25th. I bled him on the 28th; the blood was nearly as buffy as in the above cases. Circumstances required his removal to the hospital, so I saw no more of him.

I also saw, May 29, a child 15 months' old, with high fever, short breathing, and a constant severe cough. She was taken suddenly the night of May 24. I leeched her freely with the effect of cutting short the fever and cough within the first twenty-four hours.

*Dr. Newman* stated that he had now under treatment a case of pneumonia, occurring in a stout, healthy Irish boy, aged 18 years, a mechanic. He was first called to the patient on Saturday, the 2d of June. The boy returned home from his work on the Wednesday evening previous complaining of being unwell, and had not since left the house. He had taken a dose of castor oil, which had profusely purged him, and it was in a great measure in consequence of the "diarrhœa," as the parents termed it, that medical aid was sought.

At the time of visit found the patient with a hot dry skin; pulse 100; breathing hurried and laborious; pain in left side referable to the lower half of the chest; a cough, which was not, however, very severe, but accompanied with a bloody sputa.

The necessity or the propriety of venesection did not suggest itself. Calomel and Dover's powders were prescribed, every four hours, with spts. nitr. eth. ℨij., at the same intervals of time, the medicines being given alternately; and mustard applied to the sides.

The next day, the 3d, his symptoms were better. There was an abatement of the fever; a profuse diaphoresis had been established soon after commencing the treatment. The bloody sputa had lessened in quantity. The same treatment was continued, with the exception of the substitution of minute doses of tart. antimon. for the calomel, and the application of a blister on the 11th.

This morning, the 5th, the pulse is down to 85, with an entire absence of the bloody sputa and a general amelioration of all the symptoms.

The signs manifested upon auscultation, seemed to be of the same negative character as in the cases reported by Dr. Burwell. Crepitation could not be detected.

*Dr. Wyckoff* gave a case of pneumonia:

Case. John Curry, an Irishman, aged 25 years, residence Ohio street, occupation a sailor; health prior to eight months' since always good, during which time he has had intermittent fever three or four times, but has been in perfect health for the last six weeks, until last evening, when he was taken with a severe chill, followed by a difficulty of breathing; a severe headache and fever; was restless and thirsty through the night. This morning at my first visit, May 22, 1855, I find him with a pulse of 112, and full; respiration 26; skin hot and dry; face flushed; slight cough; complaining of nothing save a severe headache.

*Physical Signs.* Distinct dullness on percussion found only over the middle lobe of the right lung posteriorly. Auscultation revealed simply rude respiration.

Prescribed tart. ant. ¼ gr., in solution, every four hours, and sinapisms to the chest.

7 o'clock, P. M. Pulse 120; respiration 28. Tart. ant. had produced severe vomiting, purging, and profuse perspiration. Tart. ant. omitted, and ipecacuanha, gr. j., pulv. opii. ¼ gr., every four hours, was substituted.

May 23, 8 o'clock, A. M. Curry had slept quietly the first of the night, but was very restless the latter part; he would fall asleep frequently, but was unconscious of getting any rest. Pulse 120, and hard; respiration 32; skin hot and dry; no pain except headache; face flushed; cough slight; expectoration rusty. Dullness on percussion more marked than yesterday, anteriorly as well as posteriorly. Sonorous ronchus anteriorly and occasionally posteriorly.

Prescribed venesection in a sitting posture. After taking 12 oz. faintness was produced, two hours after which a full anodyne was given.

7 o'clock, P. M. Pulse 112, full but soft; respiration 24. Pulv. Doveri, grs v., was given, which was to be repeated, if necessary, through the night.

May 24, 8 o'clock, A. M. Pulse 120; respiration 26. Chest not examined.

Prescribed hyd. sub. mur. ipecacuanha, āā gr. j., pulv. opii, ¼ gr., every four hours.

25th. Pulse 128, rather small; respiration 28; skin hot, but moist; face flushed; cough slight; expectoration scanty, but rusty.

Calomel, opium, and ipecacuanha continued.

26th, 8 o'clock, A. M. Pulse 116; respiration 26; profuse perspiration; cough and expectoration increased, the latter still rusty. The fetor of breath and appearance of the gums clearly show that ptyalism has taken place.

Calomel discontinued. Opium and ipecac. continued.

7 o'clock, P. M. Pulse 130, and small; respiration 38; skin hot, but moist. Patient wandering and very irritable.

Prescribed decoct. of senega, with rad. glyc. and a full anodyne.

27th, 8 o'clock, A. M. Curry has had a quiet night, but is talking incoherently this morning, when not aroused. Pulse 132; respiration 32; complains of pain just inside the right nipple, which is aggravated by coughing. Dullness upon percussion, posteriorly, perceptibly increased. The sonorous ronchus may be heard occasionally, anteriorly, with an entire absence of sound posteriorly, except a slight blowing sound. The proportion of blood in expectoration increased.

At 3 o'clock, P. M., Dr. G. N. Burwell in consultation. We prescribed venesection, 16 oz., which, after standing, became very buffy. The blood was drawn in a bowl and a cup: the buff in the former was at least a quarter of an inch in thickness. The crassamentum was very firm, so much so that when a stick was passed into it it would sustain the weight of the mass.

8 o'clock, P. M. Pulse 120; respiration 26. Patient expresses himself as greatly relieved.

Pulv. Doveri. grs. vj., was prescribed, to be repeated, if necessary, every four hours through the night.

28th, 8 o'clock, A. M. Dr. G. Burwell in consultation. Pulse 112, and soft; respiration 24. Subcrepitant ronchus distinct posteriorly, and sonorous ronchus in front; skin soft and rather cool.

Prescribed *minute* doses of nitrate potassa in solution, simply as a placebo.

7 o'clock, P. M. Pulse 112, full and rather hard; respiration 24; skin hot and dry; cough slight; expectoration still streaked with blood.

Prescribed venesection, 12 oz., followed by pulv. Doveri, grs. v., to be repeated in four hours if the first did not produce rest.

29th, 8 o'clock, A. M. Dr. G. N. Burwell in consultation. Patient had rested well during the night. Pulse 92; respiration 18; skin soft and cool.

Prescribed no medicine, toast water for nourishment.

The blood after standing over night, is still much buffed, but less so than at the previous bleeding. The crassamentum is still firm.

7 o'clock, P. M. Pulse 84; respiration 18.

May 30. Pulse 80. Patient doing well.

May 31. Pulse 72. Patient wishes to sit up, and is so well that he is discharged from further treatment.

The resolutions of amendment to the constitution, proposed at the April meeting, were then brought up and passed.

*Dr. Wilcox* reported as delegate from the Association to the American Medical Association. After which adjourned to meet at Dr. Wilcox's office.

SANFORD B. HUNT, M. D., Secretary.

